# Polyamines and Hypusination Are Required for Ebolavirus Gene Expression and Replication

**DOI:** 10.1128/mBio.00882-16

**Published:** 2016-07-26

**Authors:** Michelle E. Olsen, Claire Marie Filone, Dan Rozelle, Chad E. Mire, Krystle N. Agans, Lisa Hensley, John H. Connor

**Affiliations:** a Department of Microbiology and National Emerging Infectious Disease Laboratory, Boston University, Boston, Massachusetts, USA; b Galveston National Laboratory, University of Texas Medical Branch, Galveston, Texas, USA; c U.S. Army Medical Research Institute of Infectious Diseases, and Integrated Research Facility, National Institute of Allergy and Infectious Diseases, National Institutes of Health, Fort Detrick, Maryland, USA

## Abstract

Ebolavirus (EBOV) is an RNA virus that is known to cause severe hemorrhagic fever in humans and other primates**.** EBOV successfully enters and replicates in many cell types. This replication is dependent on the virus successfully coopting a number of cellular factors. Many of these factors are currently unidentified but represent potential targets for antiviral therapeutics. Here we show that cellular polyamines are critical for EBOV replication. We found that small-molecule inhibitors of polyamine synthesis block gene expression driven by the viral RNA-dependent RNA polymerase. Short hairpin RNA (shRNA) knockdown of the polyamine pathway enzyme spermidine synthase also resulted in reduced EBOV replication. These findings led us to further investigate spermidine, a polyamine that is essential for the hypusination of eukaryotic initiation factor 5A (eIF5A). Blocking the hypusination of eIF5A (and thereby inhibiting its function) inhibited both EBOV gene expression and viral replication. The mechanism appears to be due to the importance of hypusinated eIF5A for the accumulation of VP30, an essential component of the viral polymerase. The same reduction in hypusinated eIF5A did not alter the accumulation of other viral polymerase components. This action makes eIF5A function an important gate for proper EBOV polymerase assembly and function through the control of a single virus protein.

## IMPORTANCE

Ebolavirus (EBOV) is one of the most lethal human pathogens known. EBOV requires host factors for replication due to its small RNA genome. Here we show that the host protein eIF5A in its activated form is necessary for EBOV replication. We further show that the mechanism is through the accumulation of a single EBOV protein, VP30. To date, no other host proteins have been shown to interfere with the translation or stability of an EBOV protein. Activated eIF5A is the only protein in the cell known to contain the specific modification of hypusine; therefore, this pathway is a target for drug development. Further investigation into the mechanism of eIF5A interaction with VP30 could provide insight into therapeutics to combat EBOV.

## INTRODUCTION

Ebolavirus (EBOV) and Marburg virus (MARV) are nonsegmented, negative-strand RNA viruses in the *Filoviridae* family representing two of the most lethal human pathogens known. The viruses have historically been seen in sporadic outbreaks where fatality rates range from 22 to 90% (1). The most recent EBOV outbreak that began in 2014 has illustrated our lack of understanding of viral pathogenesis and has highlighted the need for increased study of how the virus replicates. These studies can help us to understand and combat active and dormant filovirus infections.

Filoviruses are genetically simple viruses, with seven genes encoding eight proteins. With the wide array of functions required for virus replication (e.g., nucleotide, protein, and membrane syntheses), it is well accepted that these viruses require numerous host factors for replication. Host factors that contribute to filovirus infection include various attachment receptors (2), the AKT pathway (3), and Neimann-Pick C1 (membrane fusion and viral entry) (4, 5), and HSP90 and LC8 as modulators of the viral replication complex (6, 7). However, many other essential factors remain undefined.

The mammalian polyamine/hypusination pathway has been shown to play a role in the replication of several viruses (8–18). Polyamines are ubiquitous, small, basic molecules that are highly regulated by expression levels of enzymes involved in the biosynthesis pathway. Mammalian cells express three polyamines: putrescine, spermidine and spermine. Downstream of the polyamine synthesis pathway, spermidine is essential for the hypusination of eIF5A. eIF5A, the only known mammalian protein to undergo hypusination, is activated through the modification of lysine 50 to form hypusine [N^8^-(4-amino-2-hydroxybutyl)lysine] (19–21).

The mechanisms for the dependence of viral replication on polyamines and hypusination vary across viral families. For example, several viruses have polyamines present in their capsids to neutralize viral RNA (8), while in other virus infections, intracellular polyamine levels in the host cells increase (9, 10). Some viruses carry genes that encode polyamine synthetic enzymes. For example, *Chlorella* viruses contain genes encoding all the components of a complete polyamine biosynthetic pathway (12–14, 16). Furthermore, upon inhibition of polyamine synthesis, replication is decreased for both herpes simplex virus (HSV) and cytomegalovirus (CMV). For CMV specifically, polyamines are required for virus assembly, either at the level of DNA packaging or capsid envelopment (11). For HSV, polyamines are required for replication of viral DNA (15). Downstream of the polyamine synthesis pathway, activated eIF5A has been implicated in the replication of several other viruses, including dengue virus and HIV. Upon dengue virus infection of C636 cells, eukaryotic initiation factor 5A (eIF5A) (mRNA and protein) is upregulated, and inhibition of eIF5A activity resulted in increased cell death in infected cells (18). Depletion of hypusinated eIF5A (hyp-eIF5A) with drug treatment blocked HIV-1 replication by suppressing viral gene expression at the level of transcription initiation (17).

Since the polyamine synthesis and hypusination pathways have been shown to be important for the replication of several virus families, we investigated the roles of both spermidine and eIF5A during filovirus infection. Here, we show that polyamines and their role in the hypusination of eIF5A are necessary for EBOV replication, as inhibitors of these pathways prevent EBOV minigenome activity. Furthermore, depletion of polyamines through short hairpin RNA (shRNA) knockdown of spermidine synthase prevents infection with EBOV and MARV in cell culture. Last, we show that the mechanism of action is via a reduction in VP30 protein accumulation. Targeting this pathway may be a viable approach for novel EBOV therapeutics, especially given that several of the drugs utilized in this study are in clinical trials for FDA approval for other diseases.

## RESULTS

### Inhibitors of polyamine synthesis prevent EBOV gene expression.

**FIG 1:**
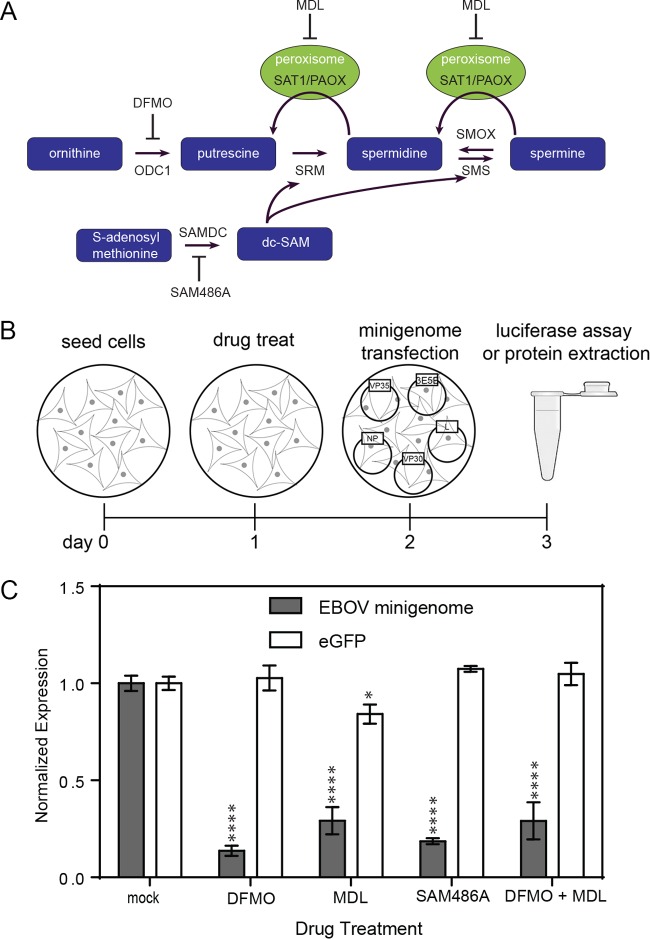
Effects of polyamine synthesis pathway inhibitors on EBOV minigenome expression. (A) Cartoon representation of the polyamine synthesis pathway. The image highlights ornithine decarboxylase (ODC) conversion of ornithine into the first polyamine putrescine. Putrescine is then converted into spermidine by spermidine synthase (SRM). Spermine synthase (SMS) then converts spermidine to spermine. *S*-Adenosylmethionine decarboxylase (SAMDC) catalyzes the conversion of SAM to decarboxy-SAM (dc-SAM), which provides the aminopropyl donor for the synthesis of both spermidine and spermine. ODC can be blocked by the irreversible inhibitor 2-difluoromethylornithine (DFMO). SAMDC is blocked by the competitive inhibitor 4-amidinoindan-1-one-2′-amidinhydrazone (SAM486A). N,N1-bis(2,3-butadienyl)-1,4-butanediamine (MDL) is an enzyme-activated irreversible inhibitor of both spermine oxidase (SMOX) and N1-acetylpolyamine oxidase (PAOX). (B) Cartoon representation of the experimental setup. The cells are seeded on day 0, treated (or mock treated) on day 1, transfected with the minigenome components on day 2, and on day 3, the cells are lysed for protein extraction or subjected to a luciferase assay. (C) EBOV minigenome-driven luciferase expression (in relative luminescence units) is shown in gray bars in the presence and absence of different inhibitors of the polyamine synthesis pathway. White bars represent EGFP expression (in relative fluorescence units) from a T7-driven plasmid, representing general gene expression in this assay. Data are normalized relative to the data for mock-treated cells. Values are means ± standard errors of the means (SEM) (error bars) from three independent experiments. Values that are significantly different from the values for mock-treated cells by Student’s *t* test are indicated by asterisks as follows: *, *P* < 0.05; ****, *P* < 0.0001.

To identify host factors necessary for EBOV replication, we investigated the effects of small-molecule inhibitors of the polyamine synthesis pathway on EBOV gene expression. The polyamine synthesis pathway is summarized in Fig. 1A. Ornithine decarboxylase (ODC) catalyzes the conversion of ornithine into the first polyamine, putrescine, and can be inhibited by the enzyme-activated irreversible inhibitor 2-difluoromethylornithine (DFMO). Putrescine is converted into spermidine by spermidine synthase (SRM). Spermine synthase (SMS) then converts spermidine to spermine. *S*-Adenosylmethionine decarboxylase (SAMDC) catalyzes the conversion of SAM to decarboxy-SAM (dc-SAM), which provides the aminopropyl donor for the synthesis of both spermidine and spermine. SAMDC can be blocked using the competitive inhibitor 4-amidinoindan-1-one-2′-amidinhydrazone (SAM486A). N,N1-bis(2,3-butadienyl)-1,4-butanediamine (MDL) is an enzyme-activated irreversible inhibitor used to inhibit both spermine oxidase (SMOX) and N1-acetylpolyamine oxidase (PAOX) (22).

Using an EBOV minigenome system (Fig. 1B and Materials and Methods) (23, 24), we tested the effects of polyamine synthesis pathway inhibitors on the expression of a *Renilla* luciferase (Rluc) reporter in BSR-T7 cells. The reporter construct contains the leader and trailer regions of the EBOV genome and is therefore under control of the EBOV polymerase. This construct is replication competent, so reporter gene expression represents both EBOV transcription and replication. Treatment of cells with DFMO or MDL to decrease the levels of free polyamines reduced expression of the minigenome reporter gene by 85% and 70%, respectively, suggesting that polyamines are necessary for reporter gene expression under the transcriptional control of the EBOV polymerase (Fig. 1C). To further elucidate the pathway, cells were treated with the compound SAM486A to block the synthesis of dc-SAM, the aminopropyl donor of spermidine and spermine. Treatment with this compound also reduced the levels of the minigenome reporter gene by 81% (Fig. 1C).

To determine whether depletion of putrescine (with DFMO) and spermidine/spermine (with MDL) had an additive effect on the reduction of reporter expression, we treated cells with both DFMO and MDL (Fig. 1C). Simultaneous treatment with both drugs showed similar levels of reporter expression to those of individual drug treatments, indicating that spermine or spermidine is necessary for EBOV transcription, while an additional depletion of putrescine does not enhance the effect. The same treatments do not prevent the expression of enhanced green flurorescent protein (EGFP) under control of T7 polymerase, indicating that the effect is specific to the viral polymerase and is not affecting T7 polymerase function or expression of host translational machinery. These results suggest that the depletion of one or more polyamine(s) interferes with EBOV gene expression.

### Spermidine synthase is necessary for EBOV infection.

To determine whether inhibitors of polyamine synthesis were preventing EBOV gene expression by specifically decreasing the levels of polyamine synthesis in the cell, we used RNA interference (RNAi) to decrease the expression of spermidine synthase (SRM), the enzyme that converts putrescine to spermidine (Fig. 1A). Because the short hairpin RNA (shRNA) constructs were developed against the human gene sequence, human cells were used for the knockdown experiments. A549 cells were transduced with three different shRNA lentivirus constructs targeting SRM or control shRNAs targeting three independent genes that do not appear to affect EBOV infection (CARS2, CCHCR1, and SH3BP5). SRM knockdown depletes the cellular pools of spermidine and spermine, decreasing the levels of available polyamines.

**FIG 2:**
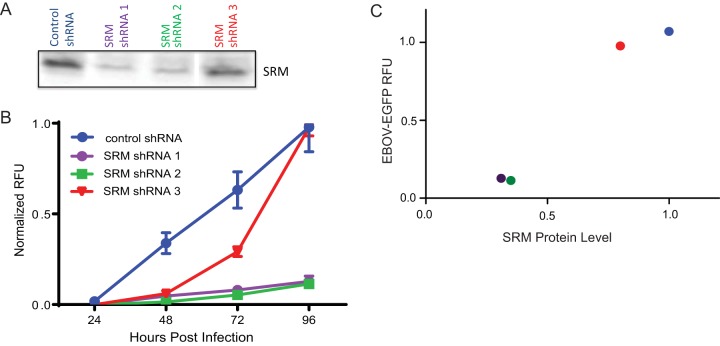
shRNA knockdown of SRM prevents EBOV-EGFP infection. (A) Immunoblot analysis of SRM protein levels following transduction of three independent shRNAs targeting SRM. Immunoblot analysis was completed 10 days posttransduction to approximate the level of SRM protein on day 4 of EBOV-EGFP infection shown in panel B (which is 10 days after transduction with shRNA). (B) Expression of EGFP from the Ebolavirus genome in control-transduced and SRM shRNA-expressing cells. Cells expressing shRNAs that target genes that do not affect EBOV infection (control shRNA) and SRM-targeting shRNAs shRNA-1, shRNA-2, and shRNA-3 are indicated. Data are normalized to control shRNA. Error bars represent standard errors of the means. RFU, relative fluorescence units. (C) Scatterplot illustrating the relative expression of Ebolavirus-expressed EGFP compared to protein levels of SRM. Data are normalized relative to control shRNA. The levels of EBOV-EGFP inhibition correlate with the percent knockdown of protein levels of SRM (Pearson correlation *R*^2^ = 0.9471; *P* = 0.0268).

Cells were transduced with several different shRNAs targeting SRM, allowed to recover for several days, and then infected with a recombinant EBOV-EGFP (EGFP-expressing EBOV) virus at a multiplicity of infection (MOI) of 0.5 (25). To approximate the levels of SRM at 4 days postinfection (dpi) with EBOV-EGFP, SRM levels were measured at 10 days posttransduction. The shRNA constructs provided variable levels of SRM reduction (Fig. 2A), where shRNA-1 and shRNA-2 reduced cellular levels of SRM compared to shRNA-3 and control shRNA. EGFP expression kinetics of cells transduced with the different shRNAs were monitored over multiple days to determine the levels of EBOV infection (Fig. 2B). These results indicated that depleted polyamine pools are detrimental to viral gene expression. At 4 dpi, EGFP levels were compared to SRM protein levels (Fig. 2C). When SRM levels were depleted by at least 50%, the amount of EGFP expressed by EBOV-EGFP also decreased by over 50% (Fig. 2C), further indicating a significant correlation between SRM protein levels and EBOV-EGFP gene expression (*R*^2^ = 0.9471; *P* = 0.0268). These data suggest that EBOV infection is dependent upon polyamine synthesis. Since EBOV minigenome reporter expression is also decreased upon inhibition of polyamine synthesis (Fig. 1C), this effect appears to be at the level of viral replication or transcription.

### Hypusination of eIF5A is necessary for EBOV gene expression.

**FIG 3:**
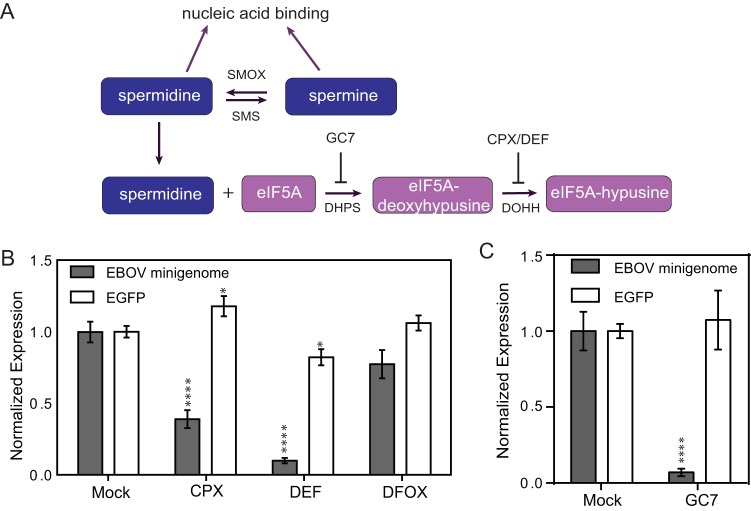
Hypusination inhibitors result in reduced EBOV minigenome expression. (A) Cartoon representation of the hypusination pathway. eIF5A is posttranslationally modified at a specific lysine residue in two reactions: (i) deoxyhypusine synthase (DHPS) transfers an aminobutyl moiety from the polyamine spermidine to lysine 50 of eIF5A; (ii) the deoxyhypusine residue is hydroxylated by deoxyhypusine hydroxylase (DOHH) to form the hypusine residue. Hypusinated eIF5A levels can be reduced by inhibiting either of the two enzymes in the pathway. DHPS can be specifically inhibited by the drug N1-guanyl-1,7-diamineheptane (GC7), and DOHH can be inhibited by the iron chelators ciclopirox (CPX) and deferiprone (DEF). (B) EBOV minigenome-driven luciferase expression (in relative luminescence units; normalized to the values for mock-treated cells) is shown in the presence and absence of different inhibitors of the hypusination pathway. EGFP expression from a T7-driven plasmid, representing general gene expression in this assay, is also shown. Values are means ± SEM (error bars) from three independent experiments. (C) EBOV minigenome-driven luciferase expression (in relative luminescence units; normalized to the values for mock-treated cells) is shown in the presence and absence of the GC7 hypusination inhibitor. EGFP expression from a T7-driven plasmid, representing general gene expression in this assay, is also shown. Values are means ± SEM (error bars) from four independent experiments. Values that are significantly different from the values for mock-treated cells by Student’s *t* test are indicated by asterisks as follows: *, *P* < 0.05; ****, *P* < 0.0001.

We next tested the hypothesis that one polyamine, spermidine, was the important polyamine required for EBOV gene expression. More specifically, we tested whether EBOV gene expression required levels of spermidine sufficient to drive the hypusination of eIF5A. Hypusination of eIF5A is completed in two steps. First, deoxyhypusine synthase (DHPS) attaches the aminobutyl group of spermidine to lysine 50 of eIF5A to form deoxyhypusinated eIF5A. The deoxyhypusine residue is then hydroxylated by deoxyhypusine hydroxylase (DOHH), forming the complete hyp-eIF5A (Fig. 3A). DOHH activity can be blocked by ciclopirox (CPX) or deferiprone (DEF), reducing hyp-eIF5A (17, 26, 27). When we tested the effects of these inhibitors on the EBOV minigenome Rluc reporter system, treatment of BSR-T7 cells with CPX and DEF resulted in a 61% and 90% reduction in Rluc expression, respectively (Fig. 3B). These results indicate that the hypusination of eIF5A is necessary for EBOV polymerase-driven reporter gene expression. An additional iron chelator, deferoxamine (DFOX), which does not block hypusination of eIF5A, had no significant effect on EBOV minigenome-driven gene expression. EGFP expression from a control plasmid was not strongly inhibited by any of these molecules, demonstrating that the effect was selective for EBOV polymerase-dependent gene expression.

To further probe the specificity of targeting the hypusination pathway, cells were treated with the DHPS inhibitor N1-guanyl-1,7-diamineheptane (GC7) (28). Treatment of BSR-T7 cells with GC7 resulted in a 91% reduction in minigenome activity (Fig. 3C), without strongly affecting expression of EGFP from a control plasmid. Together, these data support the hypothesis that the hypusination of eIF5A is specifically necessary for EBOV polymerase-driven reporter gene expression.

### Hypusination of eIF5A is necessary for EBOV and MARV infection.

**FIG 4:**
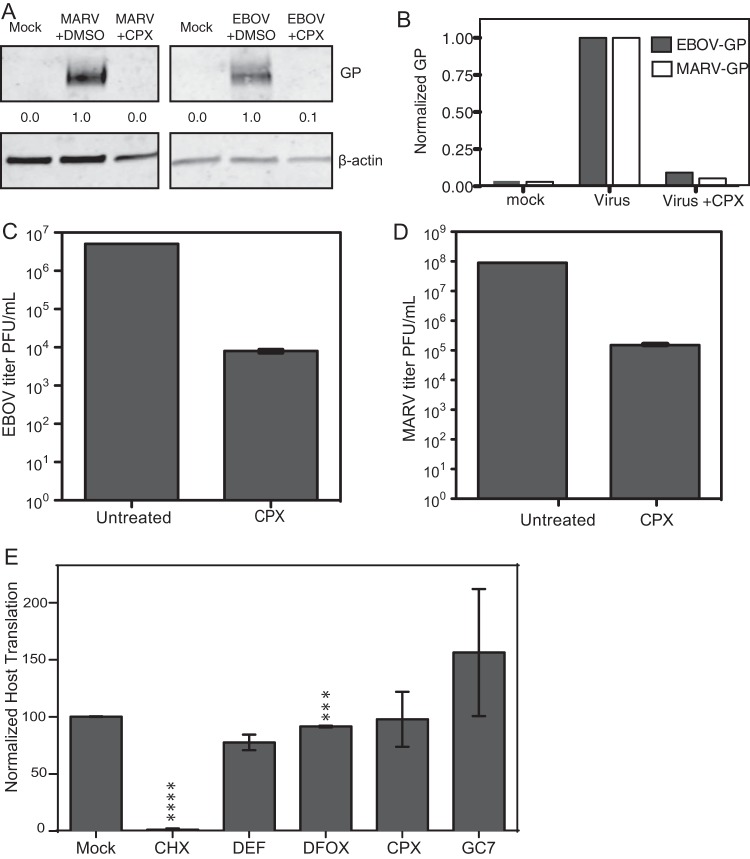
Treatment with antihypusination drug CPX reduces both EBOV and MARV infection. (A) Immunoblot against filovirus glycoprotein (GP) for MARV or EBOV, representing levels of infection in cell lysate with and without drug treatment. The numbers below the blots are the relative level of infection normalized to the β-actin loading control. (B) Graphic representation of the GP levels in panel A in drug-treated EBOV- and MARV-infected cells normalized to untreated, infected controls (Virus), and mock-infected cells. (C) Plaque assay quantification of EBOV titers in untreated versus CPX-treated cells. Supernatant was harvested and clarified, and the titers of virus in the supernatant were determined on Vero cells 72 hpi. (D) MARV titers in untreated versus CPX-treated cells, similar to panel C. (E) To assess whether drugs were affecting overall host translation, general translation of host proteins was measured in the presence and absence of various drugs using ^35^S and normalized to mock-treated values. Cycloheximide (CHX) was used as a positive control to show that host translation can be halted. Values are means ± SEM (error bars). Values that are significantly different from the values of mock-treated cells by Student’s *t* test are indicated by asterisks as follows: ***, *P* < 0.001; ****, *P* < 0.0001.

We next investigated whether the antihypusination compound CPX could decrease EBOV or MARV infection. HepG2 cells were pretreated with CPX for 24 h and then infected with EBOV at an MOI of 0.1 or MARV at an MOI of 0.5 for 72 h. When hypusination was blocked using CPX, the levels of EBOV and MARV glycoprotein (GP) expression were reduced by at least 85% when measured by immunoblotting (Fig. 4A and B). Overall infectious titers of both EBOV and MARV were also inhibited by almost 3 log units at 72 h postinfection (hpi) as measured by plaque assay (Fig. 4C and D). These results suggest that blocking hypusination of eIF5A inhibits replication of infectious EBOV as well as MARV.

### Antihypusination drugs do not significantly affect general cellular translation.

eIF5A is a translation factor that is currently thought to be important for peptide chain elongation. It is known to be essential for eukaryotic cell division as well as the translation of a subset of cellular mRNAs (29, 30). To verify that the effects of the drugs were specific to viral gene expression, and not due to an overall reduction in host translation, we measured the overall levels of translation in cells following drug treatment using [^35^S]methionine incorporation. As shown in Fig. 4E, there was a minimal effect on general cellular translation when hypusination was blocked. Together, these data indicate that general cellular translation is not affected by the inhibition of polyamine synthesis or eIF5A hypusination and the effects of reduced hyp-eIF5A are EBOV specific.

### Hypusinated eIF5A is required for VP30 protein accumulation.

**FIG 5:**
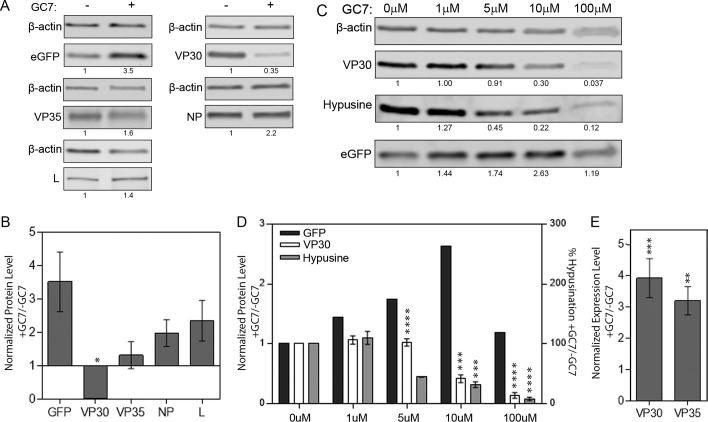
Dosage response of VP30 and hypusination to GC7. (A) Representative immunoblots for EGFP, VP30, VP35, NP, and L with β-actin loading control in the presence (+) and absence (−) of the drug (GC7). (B) Quantification of immunoblots showing relative protein levels for each of the EBOV minigenome proteins in the presence of GC7 normalized to its nontreated control. Values are means ± SEM (error bars) from three independent experiments. (C) Representative immunoblots of hypusine, VP30, and EGFP and the β-actin loading control from cells treated with increasing levels of GC7. (D) Quantification of protein levels of VP30, EGFP control (GFP), and percent hypusination (Hypusine) from cells treated with increasing micromolar concentrations of GC7. Data were normalized to nontreated control data. Values are means ± SEM (error bars) from three independent experiments. (E) RT-qPCR quantification of relative VP30 and VP35 mRNA levels (normalized to 18S rRNA) in the presence or absence of GC7. Values are means ± SEM (error bars) from four independent experiments. In panels B and D, values that are significantly different from the values for mock-treated cells (0 µM) by Student’s *t* test are indicated by asterisks as follows: *, *P* < 0.05; ***, *P* < 0.001; ****, *P* < 0.0001. In panel E, values for drug-treated cells that are significantly different from the values for untreated cells by Student’s *t* test are indicated by asterisks as follows: **, *P* < 0.01; ***, *P* < 0.001.

To gain insight into the mechanism of how hyp-eIF5A is involved in EBOV minigenome Rluc expression, we investigated whether the lack of functional eIF5A led to a decrease in the protein level of one of the components of the viral polymerase. First, we investigated the accumulation of each viral polymerase protein (expressed in the presence of all of the minigenome components) in the presence and absence of GC7. These experiments showed that there was an obvious decrease in the level of VP30 in the presence of GC7 (Fig. 5A and B). In contrast, the levels of an EGFP control increased when GC7 was added. A slight increase was also observed for the other viral components of the minigenome system: VP35, NP, and L (Fig. 5A and B). The selective decrease in VP30 levels following GC7 treatment was also seen when each of the minigenome support plasmids were transfected individually at higher concentrations (see Fig. S1 in the supplemental material). Consistent with these results, VP30 protein levels were also significantly reduced when polyamine pools were depleted with SAM486A drug treatment in BSR-T7 cells (Fig. S2A). Furthermore, these results were also reproduced in A549 cells using both GC7 and SAM486A (Fig. S2B). These results indicate that blocking polyamine synthesis and hypusination have the same effect on EBOV protein accumulation.

The decrease in VP30 protein accumulation correlated with decreased hyp-eIF5A. As seen in Fig. 5C, both VP30 and hypusine levels show a dose-dependent response to GC7 treatment. As GC7 concentrations increase, the protein levels of hyp-eIF5A and, in turn, VP30, decrease. However, the levels of the hyp-eIF5A and VP30 proteins do not decrease at the same ratio. This could indicate a threshold level of hyp-eIF5A required for VP30 protein accumulation. The reduction in protein levels seen with hyp-eIF5A and VP30 is not mimicked by the EGFP control. In fact, the opposite trend is observed. As GC7 concentrations increase, EGFP levels also increase, to a critical GC7 concentration of 10 µM (Fig. 5D).

To understand whether the lack of VP30 accumulation was due to changes in mRNA accumulation, cells were treated with GC7 to block hypusination, transfected with the minigenome support plasmids, and quantitative PCR (qRT-PCR) was performed to quantify the relative levels of VP30 and VP35 mRNA. ΔΔ*C_T_* (threshold cycle) values were calculated to compare VP30 or VP35 mRNA levels normalized to 18S, with and without drug treatment. As shown in Fig. 5E, when cells were treated with GC7 to reduce hyp-eIF5A, VP30 mRNA levels were increased (*P* = 0.0054 by the ratio paired *t* test). As a comparison, VP35 mRNA is also increased in the presence of drug (*P* = 0.0525 by the ratio paired *t* test). The data are also consistent when VP30 and VP35 are transfected into cells individually (see Fig. S3 in the supplemental material). These results indicate that a reduction in hyp-eIF5A does not reduce the transcription of EBOV genes and that the reduced accumulation of VP30 protein is not due to a reduction in VP30 mRNA.

## DISCUSSION

The data presented here demonstrate that EBOV requires polyamines for replication. When cells are treated with drugs to reduce polyamine pools, and ultimately decrease levels of hyp-eIF5A, EBOV titers and minigenome luciferase reporter gene expression are reduced. This is also seen when the hypusination pathway itself is targeted directly.

The mechanism of EBOV dependence upon polyamines and hypusinated eIF5A is unique compared to other viruses that have been reported to require these pathways. Here we identify a single viral protein requiring hypusinated eIF5A, which is subsequently required for viral gene expression. The levels of VP30 protein are significantly reduced when the hyp-eIF5A levels are decreased. This is in sharp contrast to the other components of the minigenome system, including VP35, NP, and L, where there is no significant change in protein levels in the presence of GC7. Thus, the most likely cause of the loss of viral-polymerase-driven gene expression and viral replication that is seen upon polyamine depletion and/or hypusination inhibition is through a decrease in VP30 protein accumulation. This effect is seen in multiple cell types with inhibitors of both polyamine synthesis and hypusination. Other viruses have been shown to be dependent on intact polyamine synthesis and hypusination pathways for viral transcription initiation, viral assembly, or viral replication in general (11, 15, 17, 31).

Though the precise mechanism for lower levels of VP30 accumulation are not yet clear, we hypothesize that the mechanism by which VP30 levels are reduced is through a defect in the translation of VP30 mRNA. The results described here stem from two assays: the minigenome assay, where the viral proteins are transcribed from plasmids using a T7 polymerase and then translated by host translation machinery, and the EBOV-EGFP assay, where viral mRNAs are transcribed by the viral polymerase complex and then translated by the same host translation machinery as in the minigenome system. Given that these two assays differ in the way the viral proteins are transcribed, but not in the way they are translated, yet have similar reductions in viral gene expression, we expect that the mechanism(s) causing VP30 reduction is likely to occur during translation. Furthermore, we show that the levels of VP30 mRNA are not significantly reduced by drug treatment. The data, however, do not unequivocally show that this is the only mechanism, but that it is likely contributing to reduced viral replication. There could be additional mechanisms in which polyamines and hyp-eIF5A are important for assembly or budding, which were not in the scope of this study.

Hyp-eIF5A has been defined as a translation elongation factor aiding in the processing of “hard to translate regions” such as polyproline sequences. However, the limited known functions of eIF5A do not shed much light on the mechanistic basis for VP30 dependence upon this protein. eIF5A has been shown to be directly involved in translation elongation in *Saccharomyces cerevisiae*, specifically to promote peptide bond formation between consecutive proline residues (polyproline tracts) (29, 30). It is unlikely that this role for eIF5A is directly responsible for the change in VP30 accumulation. VP30 does not contain any polyproline tracts, suggesting that the requirement for eIF5A is due to some other function. In contrast, the EBOV VP35 protein contains two PPGP sequences but is not sensitive to depletion of hyp-eIF5A. Interestingly, a recent publication reported that only a third of the eIF5A-regulated proteome contains polyproline stretches (32), implying that the presence of polyproline tracts is not the sole determinant of eIF5A dependence in protein expression.

It is possible that VP30 is not directly dependent on hyp-eIF5A but that eIF5A is modulating another protein that is important for VP30 expression. Generally, polyproline-containing proteins facilitate protein-protein interactions that function in a range of host processes (33, 34). Therefore, reducing hyp-eIF5A could decrease the translation of another specific protein necessary for the production of EBOV-VP30. Because of the relatively rapid effectiveness of eIF5A depletion on minigenome activity (less than 24 h), if the latter hypothesis is true, then the protein in question must be highly labile. A third formal possibility is that hyp-eIF5A stabilizes VP30 protein, and by reducing hyp-eIF5A, VP30 is then degraded more rapidly.

Our results demonstrate an EBOV dependence on polyamines that can limit virus replication by targeting either polyamines generally or by targeting the hypusination pathway. Future studies aim to identify the mechanism by which VP30 is sensitive to reductions in hyp-eIF5A. Potential mechanisms include translation of mRNA or protein stabilization. It is interesting to speculate why EBOV (VP30) has evolved to require hyp-eIF5A. If there is indeed a direct interaction between eIF5A and VP30 mRNA, a highly speculative hypothesis is that it is sequestering eIF5A from the translation of other cellular proteins which, in turn, may facilitate infection. Given that eIF5A is the only protein in the cell known to contain the hypusine modification, this pathway presents itself as a target for drug development through the inhibition of hypusination. Further probing into the mechanism of eIF5A modulation of VP30 levels could provide additional insight into novel therapeutics to combat this deadly disease.

## MATERIALS AND METHODS

### Cells.

BSR-T7/5 and A549 cells were cultured in Dulbecco’s modified Eagle’s medium supplemented with 10% fetal calf serum and l-glutamine (supplemented DMEM). The cells were grown in an incubator at 37°C with 5% CO_2_. HepG2 and Vero E6 cells were maintained in Eagle’s minimum essential medium (EMEM) supplemented with 10% fetal calf serum, 100 U/ml penicillin (Gibco), 100 g/ml streptomycin (Gibco), and 1% GlutaMAX (Gibco).

### Reagents.

4-[Aminoiminomethyl]-2,3-dihydro-1H-inden-1-diaminomethylene-hydrazone (SAM486A: also referred to as Sardomozide or CGP 48664) (0.4 µM in H_2_O) was provided by Novartis. N1-guanyl-1,7-diamineheptane (GC7) (10 µM in H_2_O) was purchased from LGC Biosearch Technologies. As recommended by the manufacturer, GC7 was used together in cell culture with 0.5 mM aminoguanidine to prevent destruction by monoamine oxidase (in H_2_O). Deferiprone (DEF) (250 µM in H_2_O) was purchased from Calbiochem. Ciclopirox (CPX) olamine (30 µM in H_2_O), 2-difluoromethylornithine (DFMO) (200 µM in dimethyl sulfoxide [DMSO]), and N,N1-bis(2,3-butadienyl)-1,4-butanediamine (MDL) (50 µM in DMSO) were purchased from Sigma.

The following antibodies for immunoblots were used (the sources and dilutions shown in parentheses): rabbit anti-VP30 N-terminal region (prepared by GenScript; 1:5,000), rabbit anti-hypusine (Raghavendra Mirmira, Indiana University School of Medicine [35] and EMD Millipore; 1:1,000), mouse anti-GFP (Roche; 1:1,000), mouse anti-β-actin (Santa Cruz; 1:1,000), mouse anti-VP35 6c5 (Kerafast; 1:1,000), rabbit anti-NP (Integrated Biotechnologies; 1:2,000), rabbit anti-L (Integrated Biotechnologies; 1:1,000); IRDye secondary antibodies: donkey anti-mouse 680 and donkey anti-rabbit 800 (LI-COR Biosciences; 1:10,000).

### Minigenome assay.

All minigenome assays were conducted in BSR-T7 cells, which support transfection of the multiple plasmids needed for the assay. Cells in a 24-well plate were treated with small-molecule inhibitors at the indicated concentrations (diluted in supplemented DMEM) for 24 h (with the exception of CPX, DEF, and DFOX which were administered only after transfection). Cells were then transfected with pTM1 plasmids containing the components of the EBOV polymerase complex under a T7-driven promoter in the amounts shown in the parentheses: L (115 ng), VP30 (145 ng), VP35 (115 ng), and NP (235 ng), along with a reporter construct, 3E5E (1,400 ng) encoding *Renilla* luciferase (Rluc) using Lipofectamine 3000 (Invitrogen). One hour posttransfection, drugs were added back into the transfection reaction at 2× concentration in supplemented DMEM to achieve the original dilution concentration. At 24 h posttransfection, cells were lysed with the Renilla-Glo luciferase assay system (Promega), and Rluc activity was measured using a Tecan Infinite 200 Pro multimode reader. Alternatively, the cells were lysed with NP-40 lysis buffer, and the lysates were subjected to immunoblotting. For GC7 dosage response experiments and individual plasmid transfections, 1 µg of VP30 plasmid DNA was transfected per well (24-well plate). Rluc will be expressed only if the components of the EBOV polymerase complex are expressed from the pTM1 support plasmids (VP30, VP35, NP, and L) through T7-driven transcription and translated by host translational machinery. The polymerase complex is then able to transcribe Rluc mRNA from the minigenome construct (which is flanked by the EBOV leader and trailer regions), and Rluc is subsequently translated by host machinery. The minigenome RNA template is also replicated by the polymerase complex, which amplifies reporter gene expression. The resulting reporter gene expression represents both EBOV transcription and replication.

### Immunoblots.

Cells were trypsinized and collected in NP-40 lysis buffer (Boston BioProducts) (50 mM Tris-HCl, 150 mM NaCl, 1% NP-40, and 5 mM EDTA, pH 7.4 ± 0.2) supplemented with a cocktail of protease inhibitors (Roche Complete Mini protease inhibitor cocktail). Following cell lysis, nuclear material was removed by centrifugation at 10,000 × *g* for 10 min at 4°C. Cell lysates were quantified by Bradford protein assay kit (Bio-Rad), analyzed on a denaturing Tris-HCl polyacrylamide gels, and transferred onto polyvinylidene difluoride (PVDF) membranes. Proteins of interest were detected by immunoblot analysis using primary antibodies described above and IRDye secondary antibodies and visualized using an LI-COR Odyssey CLx imaging system (LI-COR Biosciences). Quantifications of immunoblot band intensities were conducted using Fiji software (36).

### shRNA knockdown of SRM.

The spermidine synthase (SRM) knockdown experiments with pathogenic EBOV-EGFP were completed in the biosafety level 4 (BSL-4) laboratory at the U.S. Army Medical Research Institute of Infectious Diseases (USAMRIID) following approved standard operating procedures (SOPs). Lentivirus constructs (optimized for transduction in A549 cells) expressing shRNAs targeting human genes were obtained from the Broad Institute. A549 cells, seeded in 96-well plates at a low density the previous day, were transduced with shRNA lentivirus constructs to achieve an MOI of ~1. Transduction was allowed to proceed overnight, and then puromycin selection was applied for 4 days. Three shRNA constructs (sequences given in parentheses) targeted SRM: SRM shRNA-1 (CATTGGCTACTCTAGCTCGAA), SRM shRNA-2 (CATCCAAGTCTCCAAGAAGTT), and SRM shRNA-3 (CTTCATGCTGTGCAGCAAGAA). The control shRNAs (sequences given in parentheses) targeted three independent genes that do not appear to affect EBOV infection: CARS2 (CTGGCAAATCAACAGTACGTT), CCHCR1 (CTGAGTGAAGCCATTTCCAAA), and SH3BP5 (GCAACGGTGAAACTGGATGAA). After selection, the knockdown cells were infected with EBOV-EGFP at an MOI of 0.5. The relative fluorescent units (RFU) were measured daily on a SpectraMax M5 microplate reader (Molecular Devices) using GFP settings (excitation wavelength, 485 nm; emission wavelength, 515 nm; 495-nm-wavelength cutoff). Background EGFP reading from uninfected wells was subtracted from all RFU values. The data were then normalized to the averaged RFU from the controls on day 4. Immunoblotting was performed on A549 cells transduced with the SRM shRNAs or an empty vector that does not express an shRNA. The cells were selected for 10 days to approximate the level of SRM protein expression on day 4 of the EBOV-EGFP infection.

### EBOV and MARV infections with CPX treatment.

Experiments with pathogenic EBOV-EGFP and MARV were completed in the BSL-4 laboratory at the University of Texas Medical Branch (UTMB) following approved SOPs. HepG2 cells (highly susceptible to Ebolavirus infection) were seeded in 12-well plates and treated with 30 µM CPX for 24 h at 37°C and 5% CO_2_. Cells were then infected with EBOV Zaire at an MOI of 0.1 or MARV Angola at an MOI of 0.5 for 1 h followed by removal of the inoculum, four washes in phosphate-buffered saline (PBS), and the addition of fresh medium with 30 µM CPX. Supernatants were harvested and clarified at 72 h postinfection. Cell monolayers were also harvested using 2× Laemmli sample buffer (Bio-Rad) following the protocol specified by the manufacturer. The titers of the viruses in supernatant samples were then determined on Vero E6 cells using the standard plaque assay; the limit of detection was 25 PFU/ml.

### [^35^S]methionine radioactivity assay.

Pulse-labeling of HepG2 cells with [^35^S]methionine was performed as previously described (37). Cells were treated with drugs as described above for 24 h before they were washed with media lacking methionine for 1 h. Cultures were then pulsed with [^35^S]methionine (200 µCi/well EasyTag express protein labeling mix; PerkinElmer) for 45 min, lysed, and separated by sodium dodecyl sulfate-polyacrylamide gel electrophoresis (SDS-PAGE). The gel was dried and exposed to phosphor screen for 24 h before being imaged on a Bio-Rad personal molecular imager system. Total band density was quantified using ImageJ and normalized to signal from DMSO-treated cell lysates (36).

### Reverse transcriptase PCR (RT-PCR) quantitation of viral RNA.

Cells treated with GC7 for 24 h, followed by transfection with VP30, VP35, or all minigenome components were harvested 24 h posttransfection in RLT buffer (Qiagen), and total cellular RNA was purified using the RNeasy kit (Qiagen). cDNA was reverse transcribed using SuperScript II reverse transcriptase (RT) (Invitrogen) and gene-specific primers for VP30 (5′-GGT GCT GGA GGA ACT GTT AAT-3′), VP35 (5′-TGA ATG CCT CCC TAA CAC TTT-3′), and 18S rRNA (5′-CCA AGA TCC AAC TAC GAG CTT-3′) according to the protocol specified by the manufacturer. qPCR was performed using SYBR green master mix (Biotool) and gene-specific primers: VP30 (Forward [For], 5′-GAG GTG AGT ACC GTC AAT CAA G-3′; Reverse [Rev], 5′-GGT GCT GGA GGA ACT GTT AAT-3′), VP35 (For, 5′-CCA CCT GGA CCA TCA CTT TAT-3′; Rev, 5′-TGA ATG CCT CCC TAA CAC TTT-3′), and 18S rRNA (For, 5′-GGC CCT GTA ATT GGA ATG AGT C-3′; Rev, 5′-CCA AGA TCC AAC TAC GAG CTT-3′) following the manufacturer’s suggested protocol on an real-time machine (Bio-Rad CFX96RT system C1000 thermal cycler). Samples were normalized by subtracting the threshold cycle (*C_T_*) values of 18S rRNA. The fold change in viral RNA levels in drug-treated cells over non-drug-treated cells was calculated.

### Statistics.

Statistics were calculated using GraphPad Prism version 6.03 for Windows (GraphPad Software, La Jolla, CA, USA).

## SUPPLEMENTAL MATERIAL

Figure S1  VP30 protein accumulation is reduced in the presence of GC7 when individually transfected into cells. Quantification of immunoblots showing relative protein levels for each of the EBOV minigenome proteins in the presence of GC7 normalized to the value for its nontreated control when the respective protein is transfected into cells alone. Values are means ± standard errors of the means (SEM) (error bars) from three independent experiments. Download Figure S1, PDF file, 0.05 MB

Figure S2  VP30 protein accumulation is reduced in the presence of SAM486A when individually transfected into the BSR-T7 and A549 cells. (A) Quantification of immunoblots showing relative protein levels for each of the EBOV minigenome proteins in the presence of SAM486 normalized to its nontreated control when the respective protein is transfected into cells alone. Values are means ± SEM (error bars) from three independent experiments. (B) Quantification of immunoblots showing relative protein levels of VP30 in the presence of GC7 or SAM486 normalized to the value for its nontreated control in A549 cells. Values for drug-treated cells that are significantly different (*P* < 0.05) from the values for untreated cells by Student’s *t* test are indicated by an asterisk. Download Figure S2, PDF file, 0.3 MB

Figure S3  VP30 mRNA levels are not reduced with GC7 treatment when transfected individually into cells. RT-qPCR quantification of relative VP30 and VP35 mRNA levels (normalized to 18S rRNA) in the presence and absence of GC7, when individual plasmids are transfected into cells. Values are means ± SEM (error bars) from four independent experiments. The value for drug-treated cells is significantly different (*P* < 0.01) from the value for untreated cells by Student’s *t* test (indicated by two asterisks). Download Figure S3, PDF file, 0.8 MB
