# Correction for Olsen et al., “Polyamines and Hypusination Are Required for Ebolavirus Gene Expression and Replication”

**DOI:** 10.1128/mBio.01065-18

**Published:** 2018-06-05

**Authors:** Michelle E. Olsen, Claire Marie Filone, Dan Rozelle, Chad E. Mire, Krystle N. Agans, Lisa Hensley, John H. Connor

**Affiliations:** a Department of Microbiology, Boston University School of Medicine, Boston, Massachusetts, USA; b National Emerging Infectious Disease Laboratory, Boston University, Boston, Massachusetts, USA; c Galveston National Laboratory, University of Texas Medical Branch, Galveston, Texas, USA; d U.S. Army Medical Research Institute of Infectious Diseases, Fort Detrick, Maryland, USA; e Integrated Research Facility, National Institute of Allergy and Infectious Diseases, National Institutes of Health, Fort Detrick, Maryland, USA

## AUTHOR CORRECTION

Volume 7, no. 4, e00882-16, 2016, https://doi.org/10.1128/mBio.00882-16. We correct the following error in our published paper. In Materials and Methods, we described all support plasmids used in the ebolavirus (EBOV) minigenome studies as having a pTM1 backbone. Resequencing of our stocks showed that one plasmid, the VP30 plasmid used in all transfections, was a pCaggs expression vector, not a pTM1 vector. All other plasmids were verified to be pTM1 based by restriction digestion and sequencing. This led us to examine whether exchanging the pTM1-expressed VP30 for the pCaggs-expressed VP30 had any effect on our reported findings. Our findings are that VP30 mRNA expressed from the pCaggs vector accumulates and that hypusinated eIF5A is required for protein accumulation. When a pTM1-based VP30 expression vector is used, VP30 protein accumulates to the same or higher levels in the absence of hypusinated eIF5A (Fig. 1). This is the opposite phenotype observed with the pCaggs expression vector. Our results suggest that there are vector-specific sequences in pCaggs that decrease VP30 protein accumulation following the inhibition of hypusination and that the inherent coding sequence of VP30 is not the source of eIF5A dependence.

**FIG 1:**
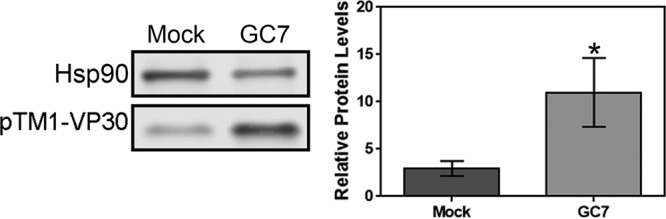
VP30 protein expressed from a pTM1 expression vector accumulates to significantly higher levels when hypusination is inhibited by GC7 treatment. (Left) Representative Western blot of VP30 protein levels and an Hsp90 loading control with and without GC7 treatment; (right) quantification of VP30 protein levels from 4 independent experiments. Error bars represent standard errors of the means. Ratio paired *t* test, *P* = 0.0143.

These results do not alter the main conclusions of our paper, namely, that blockade of polyamines or blockade of eIF5A hypusination results in a decrease in EBOV replication. These results alter our proposed mechanism by which the hypusination blockade alters EBOV replication and show that low levels of VP30 protein are not the only restriction to EBOV replication (though they likely contribute to this phenotype under the conditions that we tested). Future studies will investigate this phenomenon both in pCaggs expression vectors and in EBOV infection.

Please also note that the affiliation line should appear as shown above.

