# Supplementary figures and images for: Polyamines and Hypusination Are Required for Ebolavirus Gene Expression and Replication
Source: mBio. 2016 Jul 26;7(4):e00882-16. doi: 10.1128/mBio.00882-16 (PMC4981715; doi:10.1128/mBio.00882-16)

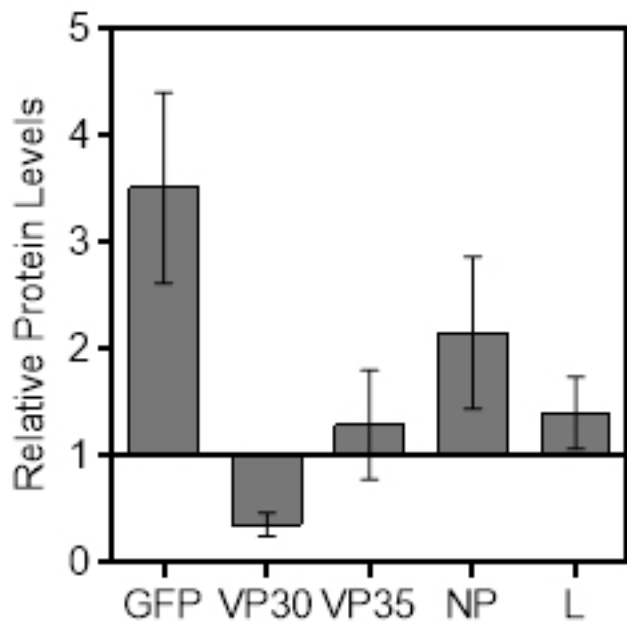

Supplement: Figure S1 — VP30 protein accumulation is reduced in the presence of GC7 when individually transfected into cells. Quantification of immunoblots showing relative protein levels for each of the EBOV minigenome proteins in the presence of GC7 normalized to the value for its nontreated control when the respective protein is transfected into cells alone. Values are means ± standard errors of the means (SEM) (error bars) from three independent experiments. Download [file mbo004162921sf1.pdf]

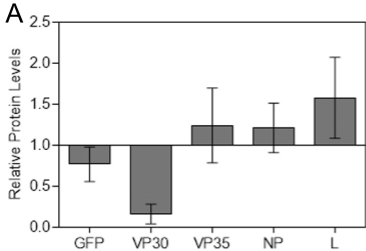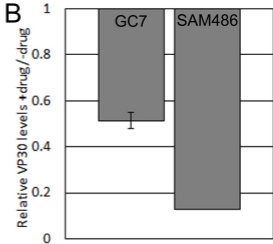

Supplement: Figure S2 — VP30 protein accumulation is reduced in the presence of SAM486A when individually transfected into the BSR-T7 and A549 cells. (A) Quantification of immunoblots showing relative protein levels for each of the EBOV minigenome proteins in the presence of SAM486 normalized to its nontreated control when the respective protein is transfected into cells alone. Values are means ± SEM (error bars) from three independent experiments. (B) Quantification of immunoblots showing relative protein levels of VP30 in the presence of GC7 or SAM486 normalized to the value for its nontreated control in A549 cells. Values for drug-treated cells that are significantly different (P < 0.05) from the values for untreated cells by Student’s t test are indicated by an asterisk. Download [file mbo004162921sf2.pdf]

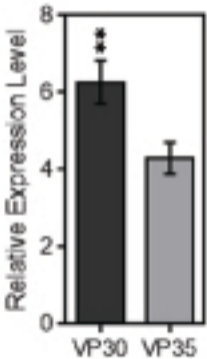

Supplement: Figure S3 — VP30 mRNA levels are not reduced with GC7 treatment when transfected individually into cells. RT-qPCR quantification of relative VP30 and VP35 mRNA levels (normalized to 18S rRNA) in the presence and absence of GC7, when individual plasmids are transfected into cells. Values are means ± SEM (error bars) from four independent experiments. The value for drug-treated cells is significantly different (P < 0.01) from the value for untreated cells by Student’s t test (indicated by two asterisks). Download [file mbo004162921sf3.pdf]
